# Co-Developing Strategies for Patient Engagement in Decentralized Rheumatic Heart Disease Care: Lessons from Northern Uganda

**DOI:** 10.5334/gh.1568

**Published:** 2026-06-23

**Authors:** Jafesi Pulle, Lisa Vaughn, Isaac Otim, Doreen Nakagaayi, Michaela Pardo, Sarah deLoizaga, McCall Miller, Joselyn Rwebembera, Emmy Okello, Nelson K. Sewankambo, Kristen Danforth, Andrea Beaton, David Watkins, James Kayima

**Affiliations:** 1 Rheumatic Heart Disease Research Collaborative, Kampala, Uganda; 2 Uganda Heart Institute, Kampala, Uganda; 3 Department of Internal Medicine, Makerere University College of Health Sciences, Kampala, Uganda; 4 Division of Emergency Medicine, Cincinnati Children’s Hospital Medical Center, Department of Pediatrics, University of Cincinnati College of Medicine, Cincinnati, Ohio, USA; 5 Department of Global Health, University of Washington, Seattle, Washington, USA; 6 College of Medicine, University of Cincinnati, Cincinnati, Ohio, USA; 7 Heart Institute, Cincinnati Children’s Hospital Medical Center, Cincinnati, Ohio, USA; 8 Department of Medicine, University of Washington, Seattle, Washington, USA

**Keywords:** rheumatic heart disease, human-centered design, co-design, ADUNU, Uganda

## Abstract

Rheumatic heart disease (RHD) requires long-term secondary antibiotic prophylaxis to prevent disease progression, yet sustained patient engagement remains challenging in many low- and middle-income countries. Within Uganda’s first integrated decentralized RHD control program, Accelerating Delivery of Rheumatic Heart Disease Preventive Services in Uganda (ADUNU), we applied a human-centered design (HCD) approach to co-develop strategies for improving engagement in care. Using two Group Level Assessment sessions (with n = 43 stakeholders) and three design focus group discussions (n = 23 stakeholders), participants identified and prioritized feasible implementation strategies. Strategies were synthesized into four domains: community integrated follow-up, structured patient health education and counselling, provider capacity and service delivery improvement, and strengthening stock planning for medicine availability. These co-developed strategies directly address barriers related to missed appointments, limited understanding of RHD, provider-patient interactions, and medicine stockouts within decentralized primary care settings. This participatory approach generated contextually grounded strategies, ready for prospective testing within decentralized RHD programs.

## Background

Rheumatic heart disease (RHD) remains a major cause of preventable cardiovascular morbidity and mortality in low- and middle-income countries (1,2). Long-term prophylaxis, delivered as monthly intramuscular benzathine penicillin G, is the only strategy proven to halt disease progression (3). The World Health Organization recommends registry-based care models to support systematic delivery and monitoring of prophylaxis for RHD (4). However, the effectiveness of registry-based RHD programs depends on sustained patient engagement and adherence, which remains challenging in many RHD endemic countries (5). Existing frameworks describe engagement as patients’ active participation in managing their health and interacting with healthcare services (6,7). Building on this, our study defines patient engagement as a dynamic health behavior reflected in observable and measurable actions, such as active self-management, adherence to prophylaxis, and retention in care.

In Uganda, the Accelerating Delivery of Rheumatic Heart Disease Preventive Services (ADUNU) program integrates RHD screening and treatment within primary health facilities (8). While decentralization of RHD care has proven feasible and improves geographic access (9), challenges persist in sustaining patient engagement. In this study, we utilized a participatory qualitative approach within the ADUNU program to generate implementable solutions to improve patient engagement.

## Methods

We applied a human-centered design (HCD) (10) approach to co-develop context-specific strategies to strengthen patient engagement in Kitgum, a predominantly rural district in northern Uganda whose young population demographic and health system characteristics are broadly representative of the country’s rural districts (11,12). As the first implementation site of Uganda’s decentralized RHD program, Kitgum serves as a learning district for program adaptation; however, no formal RHD research or control activities had been implemented in the district prior to program rollout. We employed Group Level Assessment (GLA), a structured facilitation approach that enabled stakeholders to identify problems, generate solutions, and prioritize feasible strategies (13). These sessions were followed by design focus group workshops (14). The participatory co-development process aimed to identify feasible, context-specific strategies to improve patient engagement.

We conducted two GLA sessions with patients enrolled in registry-based RHD care, family members, healthcare providers, local leaders, and village health team (VHT) members. VHTs are community-selected volunteer health workers who are not formally trained health professionals and who provide basic health education, mobilization, and linkage to healthcare services at the household level. Participants were selected to ensure diversity in age, sex, and secondary prophylaxis adherence levels among patient participants. Prompts for the GLA sessions were informed by prior qualitative work on the determinants of patient engagement within ADUNU in Kitgum. During GLAs, participants generated and prioritized potential solutions to overcome barriers to engagement in care.

Strategies were refined through three design-focused group discussions involving a selected group of stakeholders, including providers, patients, and community leaders. Individuals who participated in the GLAs were intentionally excluded from the design-focused group discussions to ensure independent perspectives and reduce the influence of prior group processes on the refinement and prioritization of strategies. Strategies were prioritized through consensus ranking based on feasibility, perceived impact, and alignment with the existing primary healthcare system. Findings were then synthesized into priority domains representing the most feasible and contextually appropriate strategies for implementation.

## Results

Two GLA sessions, involving a total of 43 participants, and three design-focused group discussions, with 23 participants, were conducted. The participatory process yielded a prioritized package of implementation strategies designed to address barriers to sustained engagement within the decentralized RHD program. In the design-focused group discussions, 18 co-generated ideas were subsequently refined and consolidated into four actionable strategy domains: community-integrated follow-up, structured patient education and counselling, provider capacity and service delivery improvement, and stock planning and medicine availability. These represent the highest-priority domains identified through stakeholder consensus based on perceived feasibility and anticipated impact (Table 1).

**Table 1:** Structured synthesis of co-generated strategies into priority implementation domains.


GROUP LEVEL ASSESSMENTS		DESIGN FOCUS GROUP DISCUSSIONS

**Initial co-generated strategies (n = 18)**	**Synthesized themes (n = 7)**	**Final priority domains (n = 4)**

Strengthen patient follow-up registers and appointment logs	Strengthening follow-up systems	**Community integrated follow-up**

Phone call reminders before clinic dates		

Provider-initiated tracing for missed visits		

Engage VHTs, peer specialists, and local leaders for tracing		

Involve family members in appointment reminders and support	Involving family members in care	

Structured counselling at diagnosis	Enhancing health education and counselling	**Structured patient education and counselling**

Ongoing education during clinic visits		

Community-based RHD education and outreach		

Use of visual and audio educational materials		

Provide structured psychosocial support platforms	Providing psychosocial support	

Train providers in communication skills	Modifying provider’s behavior through training	**Provider capacity and service delivery improvement**

Incorporate communication modules into CME		

Ensure correct injection technique to reduce pain		

Improve clinic workflow to reduce wait times	Improving service organization	

Ensure adequate consultation time for patient interaction		

Improve privacy during injection visits		

Improve forecasting of benzathine penicillin G and lidocaine	Ensuring medicine and supply availability	**Stock planning and medicine availability**

Redistribute supplies across facilities to prevent stockouts		


### 1. Community-integrated follow-up

Gaps in follow-up and retention were identified as key determinants of patient engagement. Proposed strategies included strengthening appointment registers, implementing phone-based reminders, and provider-initiated tracing for missed visits. Home-based tracing and community-linked approaches—including home involvement of VHTs, peer expert patients, family members, and local leaders—were also proposed to complement facility systems.

### 2. Structured patient education and counselling

Gaps in patient understanding and stigma were identified, and stakeholders proposed the following strategies to address these gaps: standardized counselling at diagnosis and follow-up, family involvement to support long-term care, integration of RHD education into community outreach, and consistent messaging on the purpose of long-term prophylaxis and consequences of missed doses. Simplified, visual materials were also recommended to improve comprehension across literacy levels.

### 3. Provider capacity and service delivery improvement

Provider communication and clinic workflow were identified as modifiable determinants of engagement. Improvement strategies included communication skills training within continuing medical education, reinforcement of patient-centered care, improved clinic organization to reduce waiting times and enhance privacy, and ensuring the implementation of correct injection techniques and local analgesia to address pain-related barriers.

### 4. Stock planning and medicine availability

Supply chain constraints were identified as key determinants of continuity of RHD care. Strategies to address this included routine forecasting of essential RHD commodities, proactive monitoring of facility-level stock, and redistribution mechanisms across facilities to address localized shortages. Ensuring consistent availability of antibiotics and analgesics at the point of care was emphasized to support patient engagement.

## Conclusion

Using a participatory human-centered design approach within a decentralized RHD program, stakeholders co-developed practical strategies to strengthen patient engagement across community-integrated follow-up systems, structured patient education and counseling, provider capacity and service delivery improvement, and stock planning and medicine availability. These strategies directly address common barriers in routine RHD care at the primary healthcare level, including missed visits, limited patient understanding, and inconsistent access to prophylaxis. Importantly, the proposed strategies leverage existing community and primary care structures, enhancing their feasibility within resource-constrained settings.

Consistent with prior participatory work in RHD and other chronic conditions, community involvement was central to identifying feasible solutions. This study extends previous work by translating these insights into a coherent, system-oriented package that can be implemented within primary healthcare settings.

The study’s findings should be interpreted in context: strategies were developed within a single rural district and may require adaptation in urban systems with different organizational structures. In addition, the relatively small purposely selected sample and stakeholder composition may have influenced prioritization of the final strategy domains towards more systemic solutions, which may limit transferability to other settings. Nonetheless, this study demonstrates how engaging patients, providers, and communities can generate practical and contextually relevant strategies for decentralized RHD care.

These findings provide an implementable strategy package that is ready for prospective evaluation. Future studies should assess the feasibility, acceptability, adoption, fidelity, and effectiveness of these strategies on patient engagement outcomes and explore adaptation across diverse RHD program settings. Such evaluation will be critical for determining scalability and informing integration into broader decentralized cardiovascular care programs.
